# Distinct roles of two SEC scaffold proteins, AFF1 and AFF4, in regulating RNA polymerase II transcription elongation

**DOI:** 10.1093/jmcb/mjad049

**Published:** 2023-08-01

**Authors:** Zhuanzhuan Che, Xiaoxu Liu, Qian Dai, Ke Fang, Chenghao Guo, Junjie Yue, Haitong Fang, Peng Xie, Zhuojuan Luo, Chengqi Lin

**Affiliations:** The Key Laboratory of Developmental Genes and Human Disease, School of Life Science and Technology, Southeast University, Nanjing 210096, China; The Key Laboratory of Developmental Genes and Human Disease, School of Life Science and Technology, Southeast University, Nanjing 210096, China; Co-innovation Center of Neuroregeneration, Nantong University, Nantong 226001, China; The Key Laboratory of Developmental Genes and Human Disease, School of Life Science and Technology, Southeast University, Nanjing 210096, China; The Key Laboratory of Developmental Genes and Human Disease, School of Life Science and Technology, Southeast University, Nanjing 210096, China; The Key Laboratory of Developmental Genes and Human Disease, School of Life Science and Technology, Southeast University, Nanjing 210096, China; The Key Laboratory of Developmental Genes and Human Disease, School of Life Science and Technology, Southeast University, Nanjing 210096, China; The Key Laboratory of Developmental Genes and Human Disease, School of Life Science and Technology, Southeast University, Nanjing 210096, China; School of Biological Science and Medical Engineering, Southeast University, Nanjing 210096, China; The Key Laboratory of Developmental Genes and Human Disease, School of Life Science and Technology, Southeast University, Nanjing 210096, China; Co-innovation Center of Neuroregeneration, Nantong University, Nantong 226001, China; The Key Laboratory of Developmental Genes and Human Disease, School of Life Science and Technology, Southeast University, Nanjing 210096, China; Co-innovation Center of Neuroregeneration, Nantong University, Nantong 226001, China

**Keywords:** super elongation complex, AFF1, AFF4, transcription elongation, early termination, readthrough transcription

## Abstract

The super elongation complex (SEC) containing positive transcription elongation factor b plays a critical role in regulating transcription elongation. AFF1 and AFF4, two members of the AF4/FMR2 family, act as central scaffold proteins of SEC and are associated with various human diseases. However, their precise roles in transcriptional control remain unclear. Here, we investigate differences in the genomic distribution patterns of AFF1 and AFF4 around transcription start sites (TSSs). AFF1 mainly binds upstream of the TSS, while AFF4 is enriched downstream of the TSS. Notably, disruption of AFF4 results in slow elongation and early termination in a subset of AFF4-bound active genes, whereas AFF1 deletion leads to fast elongation and transcriptional readthrough in the same subset of genes. Additionally, AFF1 knockdown increases AFF4 levels at chromatin, and *vice versa*. In summary, these findings demonstrate that AFF1 and AFF4 function antagonistically to regulate RNA polymerase II transcription.

## Introduction

In higher organisms, RNA polymerase II (Pol II) transcription is sophisticatedly regulated at multiple stages to ensure the accurate gene expression at specific temporal and spatial points (Conaway and Conaway, 2019; Cramer, 2019; Lis, 2019; Roeder, 2019). Genome-wide localization studies in metazoans have revealed that, subsequent to transcription initiation, Pol II is paused at the promoter-proximal regions of most genes (Jonkers and Lis, 2015; Core and Adelman, 2019). The release of paused Pol II into productive elongation by positive transcription elongation factor b (P-TEFb), consisting of cyclin-dependent kinase 9 (CDK9) and CCNT1/2, is a crucial rate-determining step in the transcription cycle (Marshall and Price, 1995; Wada et al., 1998; Price, 2000). The majority of P-TEFb is sequestered in the catalytically inactive 7SK small nuclear ribonucleoprotein particle (7SK snRNP) complex (Nguyen et al., 2001; Yang et al., 2001; Yik et al., 2003). Upon signal stimulation, P-TEFb is released from the inactive 7SK snRNP complex and incorporated into active complexes, such as the super elongation complex (SEC), to stimulate transcription (Bres et al., 2008; Lin et al., 2010, 2011).

SEC is composed of the ELL family members ELL/ELL2, AF9/ENL, the AF4/FMR2 family members AFF1/AFF4, and P-TEFb (Lin et al., 2010). AFF1 and AFF4 are mainly involved in the formation of SEC as the central scaffold proteins (Luo et al., 2012b). Mutations of AFF1 and AFF4 have been associated with different human diseases (Smith et al., 2011; Luo et al., 2012b; Izumi et al., 2015). SEC containing AFF4 (AFF4-SEC) efficiently induces the transcription of immediate early genes and multiple developmental genes (Lin et al., 2011). In contrast, AFF1-containing SEC (AFF1-SEC) exhibits potential in activating HIV-1 Tat transcription (Lu et al., 2014, 2015). Acute depletion of AFF4, but not AFF1, compromises the activation of HSP70 during heat shock, while simultaneous degradation of both AFF1 and AFF4 has more serious effects (Luo et al., 2012a; Lu et al., 2015; Zheng et al., 2021). However, the precise mechanisms underlying functional specificities of AFF1 and AFF4 remain unclear.

Recent studies have demonstrated that aberrant elongation rate of RNA Pol II could intervene appropriate transcription termination (Cortazar et al., 2019; Muniz et al., 2021). Pol II mutant with a slow velocity could be chased easily by the termination factor XRN2, which cleaves the nascent RNA, and thus is released from upstream of the transcription termination site (TES), while fast elongation pushes termination further downstream (Fong et al., 2015). Loss-of-function mutants of several elongation factors, including PAF1, SPT6, and CDK9, have shown similar transcription termination defects to those observed in slow Pol II mutants (Ardehali et al., 2009; Booth et al., 2018; Hou et al., 2019). Treatment with KL-1/2, the small-molecule inhibitors of SEC, destabilizes the AFF1, AFF4, and ELL2 proteins, disrupts the interaction between CCNT1 and AFF1/AFF4, and leads to a reduced Pol II elongation rate and early termination (Liang et al., 2018). However, further investigations are warranted to determine the specific roles of AFF1 and AFF4 in controlling transcription elongation.

In this study, we demonstrated distinct genomic distribution patterns of AFF1 and AFF4 around transcription start sites (TSSs) and their antagonistic roles in regulating Pol II transcription.

## Results

### Distinct distribution patterns of AFF1 and AFF4 around TSSs

**Figure 1:**
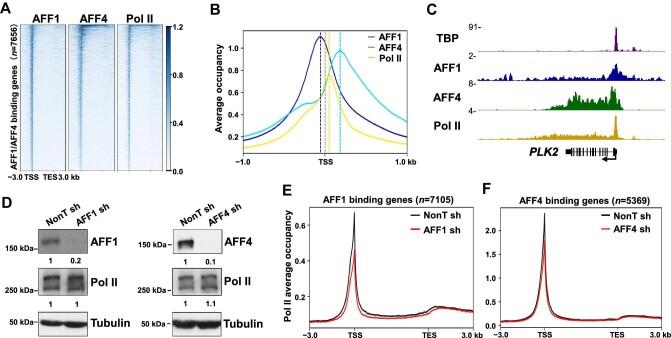
AFF1 and AFF4 exhibit diverse chromatin occupancies to regulate Pol II transcription differentially. (**A**) Heatmap showing AFF1, AFF4, and Pol II ChIP–seq signals at AFF1 and AFF4 binding genes from 3 kb upstream of the TSS to 3 kb downstream of the TES. (**B**) Metaplot analysis of AFF1, AFF4, and Pol II occupancies at AFF1 and AFF4 binding genes from 1 kb upstream to 1 kb downstream of the TSS. Grey horizontal dotted line indicates the TSS. (**C**) Genome browser tracks of the TBP, AFF1, AFF4, and Pol II occupancy profiles in the *PLK2* gene. (**D**) Western blot analysis of Pol II, AFF1, or AFF4 protein levels in A549 cells. α-Tubulin was used as a loading control. (**E** and **F**) Metagene analysis of Pol II occupancy at AFF1 binding genes (**E**) and AFF4 binding genes (**F**) from 3 kb upstream of the TSS to 3 kb downstream of the TES. The metaplots were generated by replicate 1 (rep1) ChIP–seq data.

To investigate the roles of AFF1 and AFF4 in transcriptional regulation, we first profiled their genomic occupancy patterns in A549 cells. AFF1 and AFF4 chromatin immunoprecipitation–sequencing (ChIP–seq) analyses with a false discovery rate (FDR) <0.05 identified 13078 AFF1 peaks and 6555 AFF4 peaks, respectively (Supplementary Figure S1A). The majority of AFF1 (∼65%) and AFF4 (∼74%) peaks were located at TSSs bound by Pol II (Figure 1A; Supplementary Figure S1A). Consistent with our previous report (Lin et al., 2011), AFF4 travels with Pol II into the gene body but is mostly enriched at 5′ regions of the highly transcribed genes. Interestingly, metaplot analysis suggested that AFF1 peaks are centered upstream of TSSs, while AFF4 peaks are centered downstream of TSSs (Figure 1B). The binding profiles of TATA-box binding protein (TBP), AFF1, AFF4, and Pol II at the *PLK2* gene are shown as an example (Figure 1C; Supplementary Figure S1B).

We next carried out Pol II ChIP–seq analyses in AFF1- and AFF4-depleted cells (Figure 1D; Supplementary Figure S1C). Metagene analysis indicated that AFF1 knockdown led to reduced Pol II levels at both the promoters and the transcribed genic regions, while AFF4 knockdown had minor effects on global Pol II occupancies (Figure 1E and F). Furthermore, western blot analysis revealed that total Pol II protein levels remained unchanged after AFF1 or AFF4 depletion (Figure 1D), indicating that AFF1-SEC might directly regulate Pol II genomic occupancies without affecting Pol II protein levels. Collectively, these results support that AFF1 and AFF4 might function differently.

### Depletion of AFF4 leads to slow elongation and early termination in a subset of active genes

**Figure 2:**
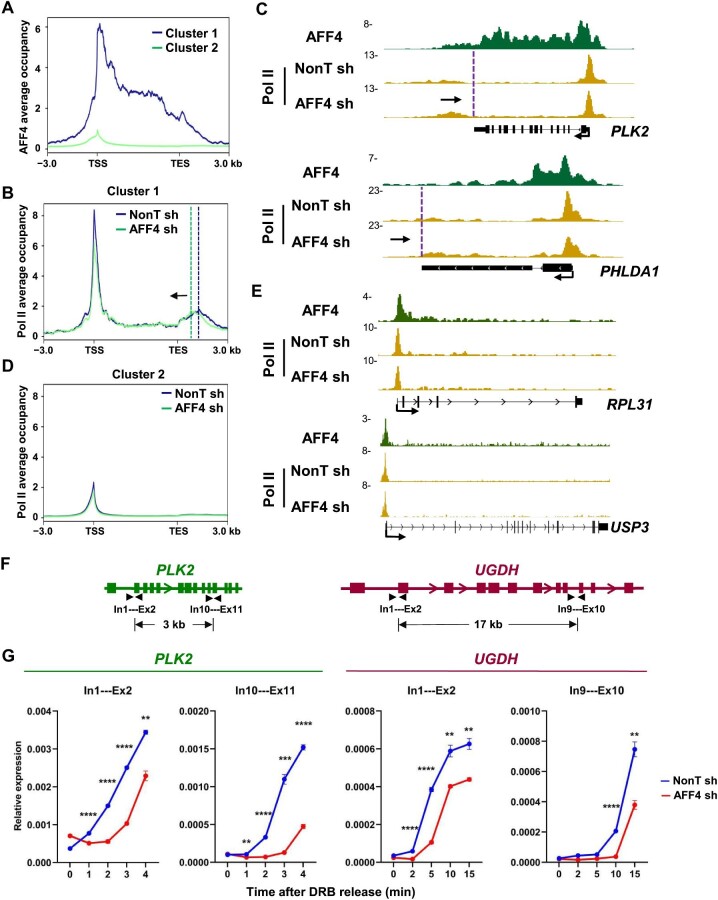
AFF4 is required for maintaining Pol II transcription elongation rate. (**A**) Metaplot analysis of AFF4 occupancies at Cluster 1 genes with strong AFF4 binding peaks (>3 kb) (*n* = 34) and Cluster 2 genes with weak AFF4 binding peaks (<3 kb) (*n* = 5287) within the region from ‒3 kb of the TSS to +3 kb of the TES in A549 cells. (**B** and **D**) Metaplots of Pol II occupancies at Cluster 1 (**B**) and Cluster 2 (**D**) genes in control and AFF4-knockdown A549 cells. Vertical dotted lines represent Pol II pause site in the termination region. Black arrow indicates the direction of Pol II peak shift. (**C** and **E**) Genome browser tracks of the AFF4 ChIP–seq signals and Pol II occupancy profiles in Cluster 1 (**C**) and Cluster 2 (**E**) genes in control and AFF4-knockdown A549 cells. (**F** and **G**) RT–qPCR analysis of pre-mRNA production in different positions of *PLK2* and *UGDH* following DRB release in control and AFF4-knockdown A549 cells. The *GAPDH* gene acts as a negative control for RT–qPCR. Data represent mean ± SEM of three biological replicates. **P < 0.01, ***P < 0.001, ****P < 0.0001, *t*-test.

We then scrutinized the effect of AFF4 on Pol II occupancy and observed two distinct gene clusters based on AFF4 occupancy signals (Figure 2A). Cluster 1 genes, including *PLK2* and *PHLDA1*, exhibited strong and broad AFF4 binding (>3 kb) and a shift of Pol II from the 3′ to 5′ end around TESs upon AFF4 knockdown (Figure 2B and C). In contrast, no significant change in Pol II occupancy signals was detected upon AFF4 knockdown in Cluster 2 genes, such as *RPL31* and *USP3*, which exhibited weak and sharp AFF4 binding (<3 kb) (Figure 2D and E).

Pol II ChIP–seq analyses indicated that AFF4 knockdown resulted in early termination in Cluster 1 genes. Since early termination was previously observed in cases where elongation rates were defective, we investigated whether Pol II elongation rate was affected after AFF4 depletion. A549 cells were treated with the transcription inhibitor 5,6-dichloro-1-β-D-ribofuranosylbenzimidazole (DRB) to inhibit transcription elongation and then cultured in fresh DRB-free medium to be released from the elongation block. The elongation rate of Pol II can be inferred from the levels of pre-mRNAs in a time-resolved manner after DRB release (Ardehali et al., 2009; Singh and Padgett, 2009; Chen et al., 2018b). The levels of pre-mRNAs from the Cluster 1 genes *PLK2* (∼6 kb) and *UGDH* (∼29 kb) in the DRB-released cells were assessed by reverse transcription–quantitative polymerase chain reaction (RT–qPCR), and both were significantly reduced after AFF4 depletion (Figure 2F and G). Thus, the depletion of AFF4 significantly reduces Pol II elongation rate.

### AFF4 knockout leads to early termination of Pol II in the serum-inducible genes

**Figure 3:**
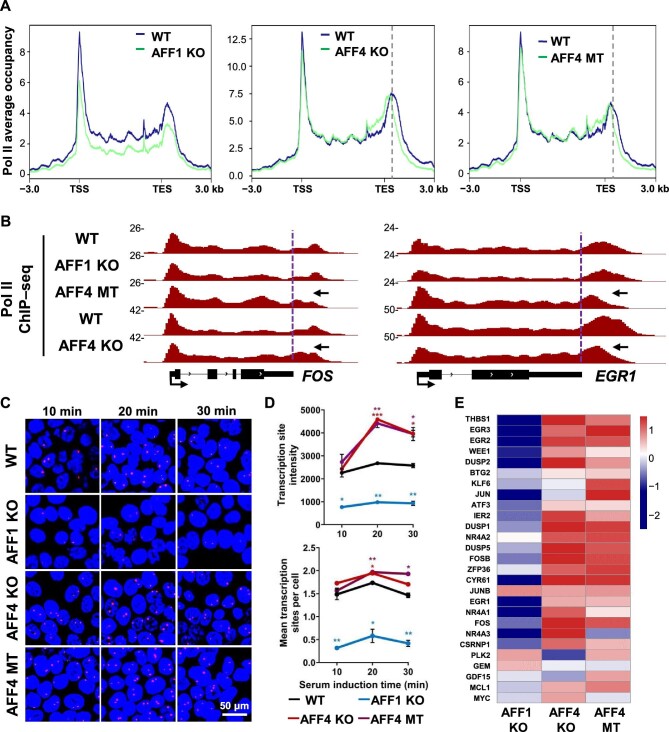
AFF4 disruption leads to early termination. (**A**) Metagene analysis comparing Pol II occupancies at the serum-inducible genes (*n* = 28) between wild-type (WT) and AFF1 KO, AFF4 KO, or AFF4 MT HCT-116 cells upon serum induction. Pol II densities are plotted in a 6-kb region (from ‒3 kb of the TSS to +3 kb of the TES) within the genes. Grey vertical dotted lines represent Pol II pause sites in the termination zone. The metaplots were generated by rep1 ChIP–seq data that were verified by ChIP–qPCR. (**B**) Genome browser tracks of Pol II ChIP–seq at two individual genes *FOS* and *EGR1* in WT, AFF1 KO, AFF4 KO, and AFF4 MT cells. Purple vertical dotted lines denote the TES and black arrows indicate Pol II peak shift toward 5′ ends. (**C** and **D**) WT, AFF1 KO, AFF4 KO, and AFF4 MT cells were subjected to serum induction for three periods. (**C**) Representative images of FOS RNA FISH showing nascent transcript. DNA was stained with DAPI in blue. (**D**) Mathematical statistics analysis of transcription site intensity and mean transcription sites per cell after serum induction. Results are representative of two biological replicates. *P < 0.05, **P < 0.01, ***P < 0.001, *t*-test. (**E**) Heatmap of two replicates of RNA-seq data generated by log_2_(fold change) shows differential expression levels of the serum-inducible genes in AFF1 KO, AFF4 KO, and AFF4 MT cells compared to WT cells.

To further examine the distinct roles of AFF1 and AFF4 in transcriptional regulation, we generated AFF1 knockout (KO) and AFF4 KO HCT116 stable cell lines using the CRISPR–Cas9 technology (Supplementary Figure S2A) and measured Pol II occupancies in the serum-inducible genes that are well characterized for studying Pol II pause release (Lin et al., 2011). Consistently, AFF1 KO led to reduced Pol II levels at both the promoters and the genic regions and consequently a reduced induction of the serum-inducible genes, whereas AFF4 KO led to early transcription termination upon serum treatment (Figure 3; Supplementary Figure S2B–F). Intriguingly, ∼30% of these genes, including *FOS, EGR1*, and *CCN1*, exhibited substantial increases in Pol II levels within the transcribing units following AFF4 KO (Figure 3B). Subsequent single-molecule RNA fluorescence *in situ* hybridization (FISH) and RNA sequencing (RNA-seq) analyses also showed a significantly increased induction of the serum-inducible genes after AFF4 KO (Figure 3C–E; Supplementary Figure S2F). Collectively, these findings suggest that AFF1 might play a role in inducing the serum-inducible genes, while AFF4 mainly functions in the transcription elongation process.

### The AFF4–P-TEFb interaction is crucial for maintaining the proper transcription elongation rate

Next, we sought to investigate the mechanism responsible for Pol II early termination after AFF4 depletion. The P-TEFb interaction domain (PID) of AFF4 is located within the first 100 amino acids of AFF4 (He et al., 2011). We here generated the PID-deleted AFF4 mutant (AFF4 MT) cell line using the CRISPR–Cas9 technique (Supplementary Figure S2G and H). IP assays confirmed that the AFF4 MT was capable of interacting with other SEC subunits but not CDK9 (Supplementary Figure S2I). Pol II ChIP–seq analysis indicated that AFF4 mutation, similar to AFF4 KO, also led to early termination (Figure 3A and B; Supplementary Figure S2E and J). Single-molecule FISH and RNA-seq analyses indeed showed a significantly increased induction of the serum-inducible genes after AFF PID deletion (Figure 3C–E; Supplementary Figure S2F). These findings indicate that the interaction between AFF4 and P-TEFb is vital for proper elongation of Pol II.

### AFF4 mutation results in the accumulation of Pol II Ser2p, Pol II Ser5p, and elongation factors in gene bodies

**Figure 4:**
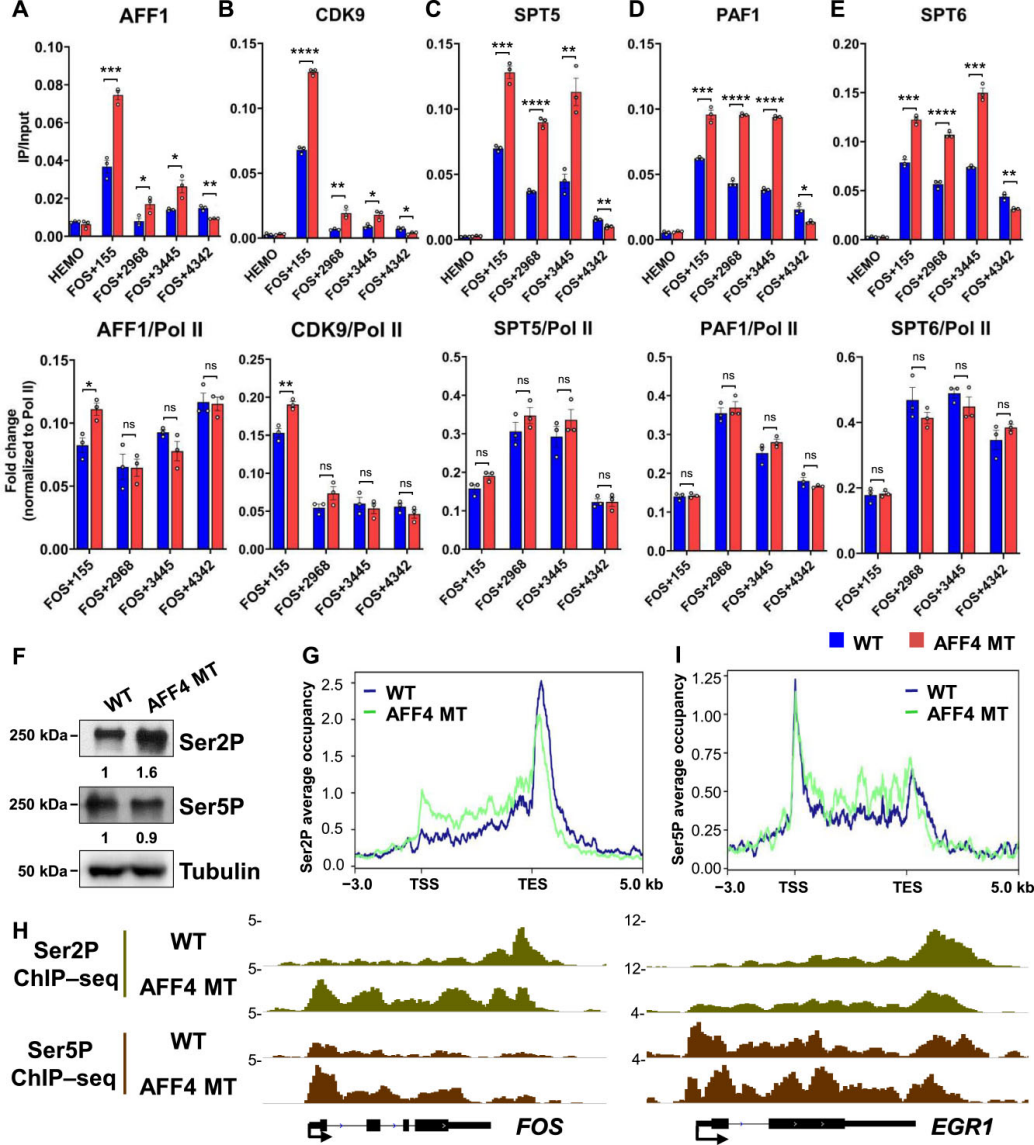
AFF4 mutation results in the accumulation of Pol II Ser2P, Pol II Ser5P, and elongation-associated factors at the 5′ end of genes. (**A**–**E**) ChIP–qPCR analysis of the occupancies (upper) and normalized occupancy changes (lower) of the elongation factors AFF1, CDK9, SPT5, PAF1, and SPT6 at the *FOS* gene in WT and AFF4 MT HCT-116 cells upon serum induction. Data are presented as mean ± SEM of three biological replicates. *P < 0.05, **P < 0.01, ***P < 0.001, ****P < 0.0001, ns = not significant, *t*-test. (**F**) The protein levels of Ser2P and Ser5P in WT and AFF4 MT HCT-116 cells. α-Tubulin was used as a loading control. (**G** and **I**) Metaplot analysis of Ser2P (**G**) and Ser5P (**I**) occupancies at the serum-inducible genes. Ser2P and Ser5P densities are plotted in the region from ‒3 kb of the TSS to +5 kb of the TES within the genes. The metaplots were generated by rep1 ChIP–seq data that were verified by ChIP–qPCR. (**H**) Genome browser tracks of Ser2P and Ser5P ChIP–seq at the serum-inducible genes *FOS* and *EGR1* in WT and AFF4 MT HCT-116 cells.

We further examined whether the occupancies of other elongation factors were also affected in AFF4 KO and AFF4 MT cells. ChIP–qPCR analyses showed that the occupancies of AFF1, CDK9, and SPT5 significantly increased at the gene body but decreased at the 3′ end of the *FOS* gene in AFF4 MT cells (Figure 4A–C). When normalized to Pol II occupancy, AFF1 and CDK9 occupancies were specifically enriched at the promoter, while SPT5 occupancy was comparable to Pol II occupancy (Figure 4A–C). Previous studies showed that depletion of PAF1 or SPT6 leads to Pol II accumulation at the 5′ end of genes and early termination (Ardehali et al., 2009; Hou et al., 2019). Interestingly, we observed increased occupancies of PAF1 and SPT6, along with Pol II, in AFF4 MT cells (Figure 4D and E). Consistently, accumulation of elongation factors in gene bodies was also detected in AFF4 KO cells (Supplementary Figure S3A). It is likely that depletion of AFF4 slows down the elongation rate of Pol II, thus leading to the accumulation of transcription elongation factors.

Previous studies reported that Pol II Ser2P accumulates at the 5′ end of genes in cells with a slow elongation rate (Fong et al., 2017). AFF4 depletion leads to early termination, resembling the phenotype of the slow Pol II elongation rate (Fong et al., 2015). Notably, we observed that protein levels of Pol II Ser2P increased dramatically in AFF4 MT cells (Figure 4F; Supplementary Figure S3B). Pol II Ser2P accumulated around TSSs and gene bodies but significantly decreased at TESs in both AFF4 MT and AFF4 KO cells (Figure 4G and H; Supplementary Figure S3C and D). Similarly, Pol II Ser5P accumulation in the 5′ region of genes was also observed (Figure 4H and I; Supplementary Figure S3C and D). Taken together, the interaction between AFF4 and P-TEFb is essential for proper Pol II elongation but dispensable for the recruitment of other SEC components and other key elongation factors like PAF1 and SPT6.

### AFF4 disruption leads to increased occupancy of CSTF2 with a 5′ shift

**Figure 5:**
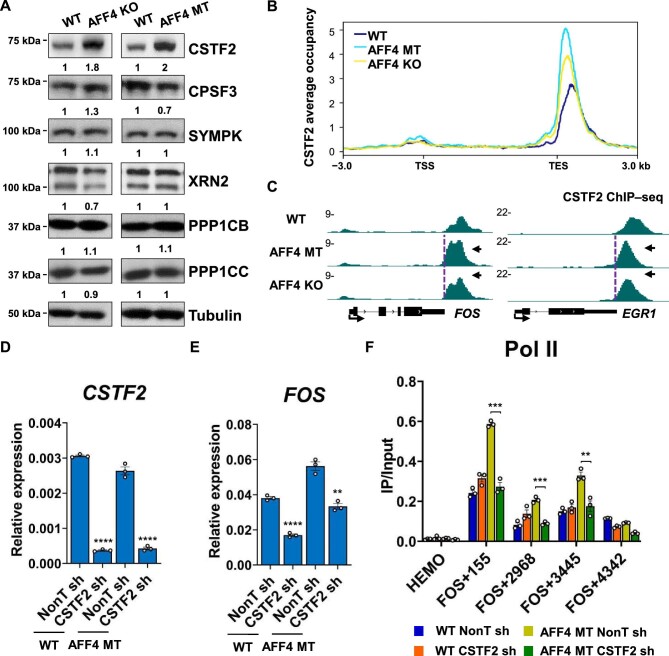
AFF4 disruption leads to increased CSTF2 occupancy with a 5′ shift. (**A**) The protein levels of CSTF2, CPSF3, SYMPK, XRN2, PPP1CB, and PPP1CC in WT, AFF4 KO, and AFF4 MT HCT-116 cells. α-Tubulin was used as a loading control. (**B**) Metaplot analysis of CSTF2 occupancies at the serum-inducible genes in WT, AFF4 KO, and AFF4 MT HCT-116 cells. CSTF2 densities are plotted in the region from −3 kb of the TSS to +3 kb of the TES within the genes. The metaplot was generated by rep1 ChIP–seq data that were verified by ChIP–qPCR. (**C**) Genome browser tracks of CSTF2 ChIP–seq at the serum-inducible genes *FOS* and *EGR1* in WT, AFF4 KO, and AFF4 MT cells. Purple vertical dotted lines denote the TES and black arrows indicate Pol II peak shift toward 5′ ends. (**D** and **E**) RT–qPCR analysis of *CSTF2* (**D**) and *FOS* (**E**) gene expression levels after CSTF2 knockdown in WT and AFF4 MT HCT-116 cells. (**F**) ChIP–qPCR analysis of Pol II occupancy at the *FOS* gene after CSTF2 knockdown in WT and AFF4 MT cells. The *HEMO* gene acts as a negative control for ChIP–qPCR. Data represent mean ± SEM of three biological replicates. **P < 0.01, ***P < 0.001, ****P < 0.0001, *t*-test.

In eukaryotes, cleavage and polyadenylation specificity factor (CPSF) and cleavage stimulation factor (CstF) are two protein complexes responsible for transcription termination of protein-coding genes (Eaton and West, 2020). We observed an obvious increase in the protein levels, but not mRNA levels, of the CstF component CSTF2 in both AFF4 KO and AFF4 MT cells, while the protein levels of other factors, such as CPSF3, SYMPK, XRN2, PPP1CB, and PPP1CC, remained unchanged (Figure 5A; Supplementary Figure S4A). ChIP–seq analysis indicated the increased CSTF2 occupancy with a 5′ shift at TESs of genes in both AFF4 KO and AFF4 MT cells (Figure 5B and C; Supplementary Figure S4B). We further depleted CSTF2 in these cells by shRNA and found that both the upregulated FOS mRNA expression and increased Pol II occupancy at the *FOS* gene were significantly compromised by CSTF2 depletion (Figure 5D–F). Interestingly, similar effects were observed by CPSF3 depletion (Supplementary Figure S4C–E). Taken together, our data demonstrate the critical role of AFF4 in maintaining proper transcription elongation and termination.

### Substantially increased AFF4 occupancies at a subset of highly active genes after AFF1 depletion

**Figure 6:**
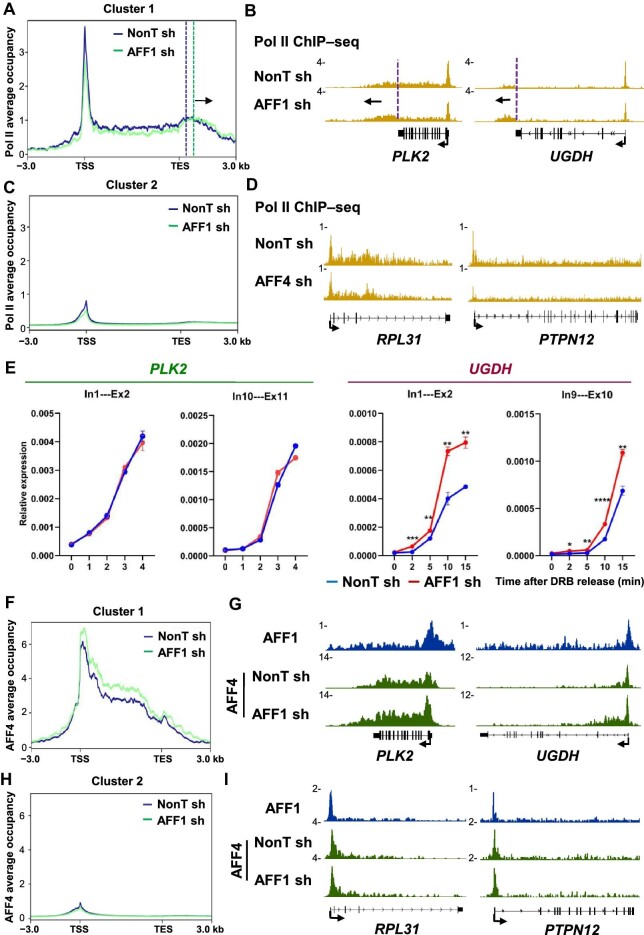
Significant increases of AFF4 occupancies at a subset of highly active genes in AFF1-depleted cells. (**A, C, F**, and **H**) Metaplots of Pol II (**A** and **C**) and AFF4 (**F** and **H**) occupancies at Cluster 1 and Cluster 2 genes within the region from −3 kb of the TSS to +3 kb of the TES in control and AFF1-knockdown A549 cells, generated by rep1 ChIP-seq data. Vertical dotted lines represent Pol II pause site in the termination region. Black arrow indicates the direction of Pol II peak shift. (**B, D, G**, and **I**) Genome browser tracks of the Pol II and AFF4 occupancy profiles in Cluster 1 (**B** and **D**) and Cluster 2 (**G** and **I**) genes in control and AFF1-knockdown A549 cells. (**E**) RT–qPCR analysis of pre-mRNA production in different positions of *PLK2* and *UGDH* following DRB release in control and AFF1-knockdown A549 cells. The *GAPDH* gene acts as a negative control for RT–qPCR. Data represent mean ± SEM of three biological replicates. *P < 0.05, **P < 0.01, ***P < 0.001, ****P < 0.0001, *t*-test.

AFF4 protein levels substantially increase after AFF1 knockdown, and *vice versa* (Supplementary Figure S5A–C). Interestingly, AFF1 knockdown led to readthrough transcription in Cluster 1 genes with strong AFF4 binding (Figure 6A and B) but only a mild decrease in Pol II occupancy at the promoters of Cluster 2 genes (Figure 6C and D). Notably, the Pol II defect in AFF1-depleted cells was reminiscent of fast Pol II phenotypes (Fong et al., 2015). RT–qPCR analyses after DRB release further revealed a quicker increase in the *UGDH* pre-mRNA level after AFF1 depletion but no obvious changes in the shorter gene *PLK2* (Figure 6E). Based on these findings, we speculated that the elongation rate and readthrough defects in AFF1-depleted cells possibly result from the increased AFF4 level. Indeed, ChIP–seq analysis confirmed the significantly increased AFF4 occupancies at Cluster 1 but not Cluster 2 genes (Figure 6F–I). Taken together, these data could point out a major role of AFF4-SEC in regulating Pol II elongation rate.

## Discussion

**Figure 7:**
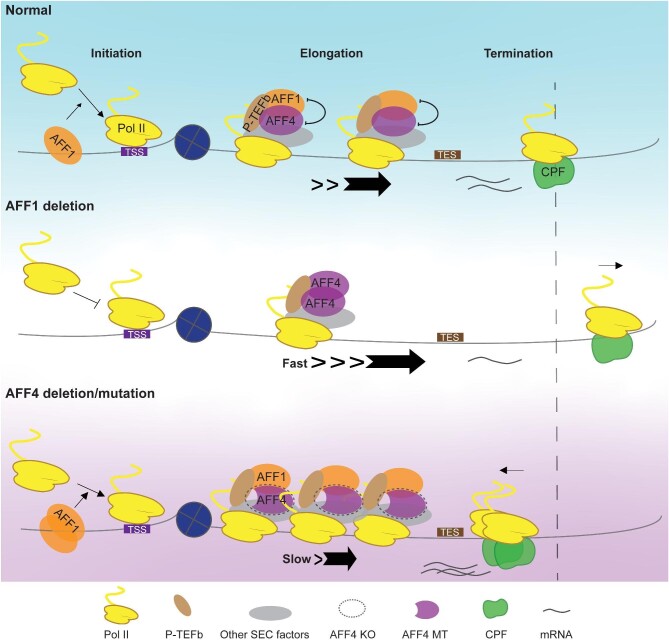
Cartoon model indicates the roles of AFF1 and AFF4 in regulating Pol II transcription. AFF1 is enriched upstream of the TSS, while AFF4 is enriched downstream of the TSS. In a subset of active genes with strong AFF4 binding signature, AFF4 disruption causes slow elongation and early termination, while AFF1 deletion increases AFF4 occupancy at chromatin, thus resulting in fast elongation and readthrough transcription.

Promoter-proximal pausing of RNA Pol II is a prevalent and critical step in eukaryotic transcription (Zhou et al., 2012; Chen et al., 2018a; Core and Adelman, 2019; Schier and Taatjes, 2020). The activity of P-TEFb is tightly controlled *in vivo* through forming different complexes, such as SEC, indicating the regulatory complexity of paused Pol II (Lu et al., 2016; Li et al., 2018; Bacon and D'Orso, 2019). Yet, the specific roles of the SEC scaffold proteins AFF1 and AFF4 in Pol II transcription elongation are still unclear. In this study, we revealed that AFF1 and AFF4 occupy upstream and downstream of the TSS, respectively. AFF1 tends to function at the transcriptional initiation stage, and depletion of AFF1 significantly reduces Pol II levels at both the promoter and the transcribing unit. AFF4 might serve as an elongation rate monitor to ensure the proper transcription elongation (Figure 7).

Substantial progress has been made in understanding the functions of SEC subunits. AFF1 and AFF4 share similar domain structure and serve as scaffolds for SEC assembly (Lin et al., 2010). Inhibiting SEC with the small-molecular inhibitors KL-1/2, which destabilize both AFF1 and AFF4, blocks the release of paused Pol II at the genome-wide scale (Liang et al., 2018). It has been proposed that AFF1 and AFF4 are able to form either heterodimer or homodimer via their C-terminal homology domains, thus regulating different subsets of genes (Yokoyama et al., 2010; Lu et al., 2015). Previous studies showed that P-TEFb within AFF4-SEC, but not AFF1-SEC, is essential for promoting the release of Pol II from pausing to active elongation at the heat shock gene *HSP70* and the *c-MYC* gene (Lin et al., 2010; Takahashi et al., 2011; Luo et al., 2012a). Our data here demonstrated that AFF1 and AFF4 co-occupy a subset of highly transcribed genes in A549 cells. However, AFF1, but not AFF4, plays a major role in promoting pause release at these genes. The reduction of Pol II levels was observed not only at the gene bodies but also at the promoter regions after depletion of AFF1. Previous study reported that the pSER domain of AFF1 is able to facilitate Pol II-mediated transcription initiation through interacting with the TBP loading factor SL1 (Okuda et al., 2015). It is likely that AFF1 could stimulate transcription at both initiation and early elongation stages via different protein complexes at the highly transcribed genes.

Interestingly, AFF4 is enriched downstream of the TSS, and depletion of AFF4 results in slow elongation and proximal termination in a subset of active genes, particularly the serum-inducible genes. Our study indicated that in contrast to AFF1, AFF4 mainly functions during the elongation stage. SEC is responsible for a rapid transcriptional induction of the serum-inducible genes. Previous studies showed that the depletion of certain elongation factors, such as PAF1 and SPT6, results in a slow elongation rate (Ardehali et al., 2009; Hou et al., 2019). However, the occupancies of these factors were not affected when AFF4 was disrupted, suggesting that AFF4 regulates elongation rate in other ways. Intriguingly, when normalizing the elongation factor occupancy to Pol II occupancy in AFF4 MT cells, we observed the increased AFF1-SEC occupancy at the *FOS* promoter. Deletion of AFF1 leads to decreased Pol II occupancy at gene promoter regions, which might account for the increased Pol II occupancy at the *FOS* gene in AFF4 KO and AFF4 MT cells.

In addition, we detected significantly incresed AFF4 protein levels after AFF1 knockdown and increased AFF1 protein levels in both AFF4 KO and AFF4 MT cells. There are two potential mechanisms. On one hand, AFF1 and AFF4 function as scaffold proteins and can assemble into separate SECs. Our study suggests a competitive relationship between AFF1 and AFF4, in which both proteins compete for binding to other SEC components. When one protein is depleted, the other one increases its association with these SEC components, thus stabilizing protein levels. On the other hand, recent study has reported that inhibition of CDK9 promotes wild-type p53 stability through phosphorylating MDM2 to reduce the MDM2-mediated ubiquitination and degradation of wild-type p53 and phosphorylating SIRT1 to suppress its deacetylase activity, respectively (Yao et al., 2022). In our study, AFF4–CDK9 might influence the stability of AFF1 protein via the phosphorylation of the E3 ubiquitin ligase or acetyltransferase, and *vice versa*, although the specific mechanism requires further investigation.

In AFF4 KO and AFF4 MT cells, we observed a clear 5′ switch of Pol II and termination factors, such as CSTF2, at the serum-inducible genes upon serum treatment, indicating that AFF4 regulates transcription depending on its interaction with P-TEFb to a large extent. AFF4 KO and AFF4 mutation increase the CSTF2 protein level without affecting its RNA level but accompanied with an increase in CSTF2 occupancy at genes. CSTF can bind to the C-terminal domain of Pol II during transcription and be recruited to chromatin for 3′ end processing (Kuehner et al., 2011). It is likely that increased Pol II levels at termination regions lead to the increased CSTF2 occupancy, thereby stabilizing the CSTF2 protein. Another possible explanation is that slow Pol II elongation rate could lead to increased cross-linking efficiencies of termination factors with chromatin. However, we also cannot exclude that AFF4-SEC may directly regulate the protein stability and chromatin recruitment of CSTF2 and other termination factors. Further investigation is required to determine the exact mechanism underlying early termination defects after AFF4 depletion.

## Materials and methods

### Cell lines and culture conditions

A549, HEK293T, and HCT-116 (WT, AFF1 KO, AFF4 KO, and AFF4 MT) cells were cultured in Dulbecco’s modified Eagle’s medium (DMEM; HyClone), supplemented with 10% fetal bovine serum (FBS; ExCell Bio) and 1% penicillin–streptomycin (HyClone), at 37°C with 5% CO_2_ in a humidified incubator and routinely passaged 2–3 times a week.

### Plasmid construction

Human AFF1 and AFF4 sgRNA oligos were subcloned into lentiCRISPR v2 vector using *Bbs*1 sites. Human AFF1, AFF4, CSTF2, and CPSF3 shRNA oligos were subcloned into pLKO.1 vector using *Age*1/*Eco*R1 sites.

### Generation of stable cell lines

To generate AFF1 KO and AFF4 KO cell lines, HEK293T cells were co-transfected with two sgRNA plasmids, psPAX2 packaging plasmids, and pMD2.G envelope plasmids using Lipofectamine 2000 (Thermo Fisher Scientific) according to the manufacturer's protocol. After 16 h, the medium was replaced with fresh medium. After 48 and 72 h, the lentiviral supernatant was collected and filtered through a 0.45-μm filter. HCT-116 cells were infected with the filtered lentiviral supernatant together with polybrene (Sigma) at a concentration of 8 μg/ml. After 24 h, the infected cells were subjected to selection with 2 μg/ml puromycin for 7 days. The selective medium was refreshed every 4 days. After that, colonies were washed with phosphate-buffered saline (PBS), picked using yellow tips, and transferred to a 48-well plate containing selective medium. When growing full, half of the cells were used for extracting genomic DNA and the other half were frozen.

### Serum induction

HCT-116 cells were cultured in DMEM supplemented with 10% FBS. For serum starvation, the cells were washed twice with PBS, transferred to DMEM without serum, and then incubated for 40 h at 37°C. Afterwards, the medium was replaced with preheated complete medium, and the cells were further incubated for 30 min at 37°C. Finally, the cells were collected for subsequent experiments.

### IP and western blot analysis

Cells were harvested in cold PBS, pelleted by centrifugation at 1000 rpm for 5 min at 4°C, and lysed in high-salt buffer containing 20 mM HEPES (pH 7.4), 1 mM MgCl_2_, 10 mM KCl, and 420 mM NaCl with protease inhibitor cocktail (Sigma–Aldrich) for 30 min at 4°C. The lysate was centrifugated at 14800 rpm for 15 min at 4°C. The supernatant was diluted with cold PBS to reduce the concentration of NaCl to 200 mM and incubated with protein A beads and antibody for 5 h at 4°C. Then, the beads were washed three times with wash buffer and boiled with sodium dodecyl sulfate (SDS). The eluted proteins were subjected to SDS–polyacrylamide gel electrophoresis and transferred to the polyvinylidene fluoride membrane for western blot analysis.

### RNA-seq

Total RNA was extracted using the RNeasy kit (QIAGEN) according to the instructions of the manufacturer. RNA was treated with DNase I (NEB) and re-purified on the column. Reverse transcription was performed using PrimeScript^™^ RT Master Mix (TaKaRa). cDNA was amplified using iTaq^™^ Universal SYBR^®^ Green Supermix (Bio-Rad) on CFX96 (Bio-Rad). Housekeeping gene *GAPDH* was used as the normalization control. To generate an RNA-seq library, total RNA was polyadenylated, fragmented, and reverse-transcribed. Then, reversed cDNA was ligated to adapters for sequencing (Illumina). Three independent biological replicates were performed.

### ChIP–seq

ChIP was performed according to the previously published protocol (Guo et al., 2020). Briefly, A549 and HCT-116 cells were cross-linked with 1% formaldehyde for 10 min and quenched with glycine for 5 min at room temperature. Fixed cells were sonicated and immunoprecipitated with the mixture of beads and antibody overnight at 4°C. ChIP-DNA was purified using a PCR purification kit (QIAGEN) for qPCR using iTaq™ Universal SYBR Green Supermix (Bio-Rad) on CFX96 (Bio-Rad). The ChIP–seq sample preparation kit was used to generate a ChIP–seq library. Three independent biological replicates (rep1, rep2, and rep3) were performed.

### Antibodies

AFF1, AFF4, CDK9, ENL, and Pol II antibodies were homemade (Lin et al., 2010; Yue et al., 2021). Pol II Ser2P (ab5095), Pol II Ser5P (ab5131), and PAF1 (ab20662) rabbit polyclonal antibodies were purchased from Abcam; SPT5 (A9193), SPT6 (A16434), CSTF2 (A8116), CPSF3 (A12368), SYMPK (A8722), XRN2 (A18350), PPP1CB (A13528), and PPP1CC (A4025) rabbit polyclonal antibodies were purchased from ABclonal.

### RNA FISH and data analysis

FOS RNA FISH was performed according to the previously published protocol (Guo et al., 2020). Briefly, cells were seeded on 12-mm round coverslips in a 12-well plate, subjected to serum induction, fixed by 3.7% formaldehyde, and permeabilized by 70% ethanol. After washing by wash buffer A, the cells were hybridized with FISH probes in hybridization buffer for 4 h at 37°C. Then, the coverslips were mounted by mounting buffer and images were taken under a Zeiss LSM 700 confocal microscope.

### DRB treatment and RT–qPCR

Cells were treated with 100 μM DRB to inhibit Pol II transcription elongation. After incubation for 3 h at 37°C, the cells were washed with PBS three times and then incubated with warm medium for different time points. The cells were lysed in TRIzol reagent (Vazyme) for total RNA extraction, and reverse transcription was performed using ABScript III RT Master Mix (ABclonal). The *GAPDH* gene acts as a negative control for RT–qPCR. Three biological replicates were performed.

### RNA-seq analysis

Clean reads were subjected to the TopHat and Cufflinks pipelines based on the human genome (UCSC Genome, GRCh37/hg19) (Trapnell et al., 2009, 2010, 2012). The Cuffdiff program within Cufflinks was used to test statistically significant differences in gene expression levels between wild-type and AFF1 KO, AFF4 KO, or AFF4 MT cells.

### ChIP–seq analysis

TBP ChIP–seq data in HepG2 cells were downloaded from Gene Expression Omnibus (GEO) GSE31477. AFF1, AFF4, and Pol II ChIP–seq data in A549 cells and Pol II, Pol II Ser2P, Pol II Ser5P, and CSTF2 ChIP–seq data in serum-treated HCT-116 cells were generated in this study and acquired through the default Illumina pipeline using Casava v1.8. Clean reads were aligned to the human genome (UCSC Genome, GRCh37/hg19) using Bowtie2 (v2.2.5) with default mode (Langmead and Salzberg, 2012). Peak calling was performed with MACS2 (v2.1.1) (Zhang et al., 2008). For AFF1 and AFF4 peaks, associated control samples were used to determine statistical enrichment at *P* < 1e−8 and FDR < 0.05. For Pol II peaks, enrichments were determined at *P* < 1e−8 and FDR < 0.05. The ChIP–seq enrichment is displayed as reads per million. Gene annotations and TSS information were from the human genome (UCSC Genome, GRCh37/hg19) by the R package ChIPseeker (Yu et al., 2015). Gene Ontology analysis was performed with DAVID (Huang et al., 2009).

## Supplementary Material

mjad049_Supplemental_File

## Data Availability

ChIP–seq and RNA-seq data generated in this study have been deposited at GEO under the accession number GSE207813. TBP ChIP–seq data were downloaded from GEO with the accession number GSE31477.

## References

[bib1] Ardehali M.B., Yao J., Adelman K. et al. (2009). Spt6 enhances the elongation rate of RNA polymerase II in vivo. EMBO J. 28, 1067–1077. 19279664 10.1038/emboj.2009.56 PMC2683705

[bib2] Bacon C.W., D'Orso I. (2019). CDK9: a signaling hub for transcriptional control. Transcription 10, 57–75. 30227759 10.1080/21541264.2018.1523668 PMC6602564

[bib3] Booth G.T., Parua P.K., Sanso M. et al. (2018). Cdk9 regulates a promoter-proximal checkpoint to modulate RNA polymerase II elongation rate in fission yeast. Nat. Commun. 9, 543. 29416031 10.1038/s41467-018-03006-4 PMC5803247

[bib4] Bres V., Yoh S.M., Jones K.A. (2008). The multi-tasking P-TEFb complex. Curr. Opin. Cell Biol. 20, 334–340. 18513937 10.1016/j.ceb.2008.04.008 PMC2628440

[bib5] Chen F.X., Smith E.R., Shilatifard A. (2018a). Born to run: control of transcription elongation by RNA polymerase II. Nat. Rev. Mol. Cell Biol. 19, 464–478. 29740129 10.1038/s41580-018-0010-5

[bib6] Chen X., Zhang J.X., Luo J.H. et al. (2018b). CSTF2-induced shortening of the RAC1 3′UTR promotes the pathogenesis of urothelial carcinoma of the bladder. Cancer Res. 78, 5848–5862. 30143523 10.1158/0008-5472.CAN-18-0822

[bib7] Conaway R.C., Conaway J.W. (2019). The hunt for RNA polymerase II elongation factors: a historical perspective. Nat. Struct. Mol. Biol. 26, 771–776. 31439940 10.1038/s41594-019-0283-1

[bib8] Core L., Adelman K. (2019). Promoter-proximal pausing of RNA polymerase II: a nexus of gene regulation. Genes Dev. 33, 960–982. 31123063 10.1101/gad.325142.119 PMC6672056

[bib9] Cortazar M.A., Sheridan R.M., Erickson B. et al. (2019). Control of RNA pol II speed by PNUTS–PP1 and Spt5 dephosphorylation facilitates termination by a ‘sitting duck torpedo’ mechanism. Mol. Cell 76, 896–908.e4. 31677974 10.1016/j.molcel.2019.09.031 PMC6927536

[bib10] Cramer P. (2019). Organization and regulation of gene transcription. Nature 573, 45–54. 31462772 10.1038/s41586-019-1517-4

[bib11] Eaton J.D., West S. (2020). Termination of transcription by RNA polymerase II: BOOM! Trends Genet. 36, 664–675. 32527618 10.1016/j.tig.2020.05.008

[bib12] Fong N., Brannan K., Erickson B. et al. (2015). Effects of transcription elongation rate and Xrn2 exonuclease activity on RNA polymerase II termination suggest widespread kinetic competition. Mol. Cell 60, 256–267. 26474067 10.1016/j.molcel.2015.09.026 PMC4654110

[bib13] Fong N., Saldi T., Sheridan R.M. et al. (2017). RNA pol II dynamics modulate co-transcriptional chromatin modification, CTD phosphorylation, and transcriptional direction. Mol. Cell 66, 546–557.e3. 28506463 10.1016/j.molcel.2017.04.016 PMC5488731

[bib14] Guo C., Che Z., Yue J. et al. (2020). ENL initiates multivalent phase separation of the super elongation complex (SEC) in controlling rapid transcriptional activation. Sci. Adv. 6, eaay4858. 32270036 10.1126/sciadv.aay4858 PMC7112754

[bib15] He N., Chan C.K., Sobhian B. et al. (2011). Human polymerase-associated factor complex (PAFc) connects the super elongation complex (SEC) to RNA polymerase II on chromatin. Proc. Natl Acad. Sci. USA 108, E636–E645. 21873227 10.1073/pnas.1107107108 PMC3169135

[bib16] Hou L., Wang Y., Liu Y. et al. (2019). Paf1C regulates RNA polymerase II progression by modulating elongation rate. Proc. Natl Acad. Sci. USA 116, 14583–14592. 31249142 10.1073/pnas.1904324116 PMC6642404

[bib17] Huang W., Sherman B.T., Lempicki R.A. (2009). Systematic and integrative analysis of large gene lists using DAVID bioinformatics resources. Nat. Protoc. 4, 44–57. 19131956 10.1038/nprot.2008.211

[bib18] Izumi K., Nakato R., Zhang Z. et al. (2015). Germline gain-of-function mutations in AFF4 cause a developmental syndrome functionally linking the super elongation complex and cohesin. Nat. Genet. 47, 338–344. 25730767 10.1038/ng.3229 PMC4380798

[bib19] Jonkers I., Lis J.T. (2015). Getting up to speed with transcription elongation by RNA polymerase II. Nat. Rev. Mol. Cell Biol. 16, 167–177. 25693130 10.1038/nrm3953 PMC4782187

[bib20] Kuehner J.N., Pearson E.L., Moore C. (2011). Unravelling the means to an end: RNA polymerase II transcription termination. Nat. Rev. Mol. Cell Biol. 12, 283–294. 21487437 10.1038/nrm3098 PMC6995273

[bib21] Langmead B., Salzberg S.L. (2012). Fast gapped-read alignment with Bowtie 2. Nat. Methods 9, 357–359. 22388286 10.1038/nmeth.1923 PMC3322381

[bib22] Li Y., Liu M., Chen L.F. et al. (2018). P-TEFb: finding its ways to release promoter-proximally paused RNA polymerase II. Transcription 9, 88–94. 28102758 10.1080/21541264.2017.1281864 PMC5834220

[bib23] Liang K., Smith E.R., Aoi Y. et al. (2018). Targeting processive transcription elongation via SEC disruption for MYC-induced cancer therapy. Cell 175, 766–779.e17. 30340042 10.1016/j.cell.2018.09.027 PMC6422358

[bib24] Lin C., Garrett A.S., De Kumar B. et al. (2011). Dynamic transcriptional events in embryonic stem cells mediated by the super elongation complex (SEC). Genes Dev. 25, 1486–1498. 21764852 10.1101/gad.2059211 PMC3143939

[bib25] Lin C., Smith E.R., Takahashi H. et al. (2010). AFF4, a component of the ELL/P-TEFb elongation complex and a shared subunit of MLL chimeras, can link transcription elongation to leukemia. Mol. Cell 37, 429–437. 20159561 10.1016/j.molcel.2010.01.026 PMC2872029

[bib26] Lis J.T. (2019). A 50 year history of technologies that drove discovery in eukaryotic transcription regulation. Nat. Struct. Mol. Biol. 26, 777–782. 31439942 10.1038/s41594-019-0288-9 PMC7106917

[bib27] Lu H., Li Z., Xue Y. et al. (2014). AFF1 is a ubiquitous P-TEFb partner to enable Tat extraction of P-TEFb from 7SK snRNP and formation of SECs for HIV transactivation. Proc. Natl Acad. Sci. USA 111, E15–E24. 24367103 10.1073/pnas.1318503111 PMC3890890

[bib28] Lu H., Li Z., Zhang W. et al. (2015). Gene target specificity of the super elongation complex (SEC) family: how HIV-1 Tat employs selected SEC members to activate viral transcription. Nucleic Acids Res. 43, 5868–5879. 26007649 10.1093/nar/gkv541 PMC4499153

[bib29] Lu X., Zhu X., Li Y. et al. (2016). Multiple P-TEFbs cooperatively regulate the release of promoter-proximally paused RNA polymerase II. Nucleic Acids Res. 44, 6853–6867. 27353326 10.1093/nar/gkw571 PMC5001612

[bib30] Luo Z., Lin C., Guest E. et al. (2012a). The super elongation complex family of RNA polymerase II elongation factors: gene target specificity and transcriptional output. Mol. Cell. Biol. 32, 2608–2617. 22547686 10.1128/MCB.00182-12 PMC3434493

[bib31] Luo Z., Lin C., Shilatifard A. (2012b). The super elongation complex (SEC) family in transcriptional control. Nat. Rev. Mol. Cell Biol. 13, 543–547. 22895430 10.1038/nrm3417

[bib32] Marshall N.F., Price D.H. (1995). Purification of P-TEFb, a transcription factor required for the transition into productive elongation. J. Biol. Chem. 270, 12335–12338. 7759473 10.1074/jbc.270.21.12335

[bib33] Muniz L., Nicolas E., Trouche D. (2021). RNA polymerase II speed: a key player in controlling and adapting transcriptome composition. EMBO J. 40, e105740. 34254686 10.15252/embj.2020105740 PMC8327950

[bib34] Nguyen V.T., Kiss T., Michels A.A. et al. (2001). 7SK small nuclear RNA binds to and inhibits the activity of CDK9/cyclin T complexes. Nature 414, 322–325. 11713533 10.1038/35104581

[bib35] Okuda H., Kanai A., Ito S. et al. (2015). AF4 uses the SL1 components of RNAP1 machinery to initiate MLL fusion- and AEP-dependent transcription. Nat. Commun. 6, 8869. 26593443 10.1038/ncomms9869 PMC4673504

[bib36] Price D.H. (2000). P-TEFb, a cyclin-dependent kinase controlling elongation by RNA polymerase II. Mol. Cell. Biol. 20, 2629–2634. 10733565 10.1128/mcb.20.8.2629-2634.2000 PMC85478

[bib37] Roeder R.G. (2019). 50+ years of eukaryotic transcription: an expanding universe of factors and mechanisms. Nat. Struct. Mol. Biol. 26, 783–791. 31439941 10.1038/s41594-019-0287-x PMC6867066

[bib38] Schier A.C., Taatjes D.J. (2020). Structure and mechanism of the RNA polymerase II transcription machinery. Genes Dev. 34, 465–488. 32238450 10.1101/gad.335679.119 PMC7111264

[bib39] Singh J., Padgett R.A. (2009). Rates of in situ transcription and splicing in large human genes. Nat. Struct. Mol. Biol. 16, 1128–1133. 19820712 10.1038/nsmb.1666 PMC2783620

[bib40] Smith E., Lin C., Shilatifard A. (2011). The super elongation complex (SEC) and MLL in development and disease. Genes Dev. 25, 661–672. 21460034 10.1101/gad.2015411 PMC3070929

[bib41] Takahashi H., Parmely T.J., Sato S. et al. (2011). Human mediator subunit MED26 functions as a docking site for transcription elongation factors. Cell 146, 92–104. 21729782 10.1016/j.cell.2011.06.005 PMC3145325

[bib42] Trapnell C., Pachter L., Salzberg S.L. (2009). TopHat: discovering splice junctions with RNA-Seq. Bioinformatics 25, 1105–1111. 19289445 10.1093/bioinformatics/btp120 PMC2672628

[bib43] Trapnell C., Roberts A., Goff L. et al. (2012). Differential gene and transcript expression analysis of RNA-seq experiments with TopHat and Cufflinks. Nat. Protoc. 7, 562–578. 22383036 10.1038/nprot.2012.016 PMC3334321

[bib44] Trapnell C., Williams B.A., Pertea G. et al. (2010). Transcript assembly and quantification by RNA-Seq reveals unannotated transcripts and isoform switching during cell differentiation. Nat. Biotechnol. 28, 511–515. 20436464 10.1038/nbt.1621 PMC3146043

[bib45] Wada T., Takagi T., Yamaguchi Y. et al. (1998). Evidence that P-TEFb alleviates the negative effect of DSIF on RNA polymerase II-dependent transcription in vitro. EMBO J. 17, 7395–7403. 9857195 10.1093/emboj/17.24.7395 PMC1171084

[bib46] Yang Z., Zhu Q., Luo K. et al. (2001). The 7SK small nuclear RNA inhibits the CDK9/cyclin T1 kinase to control transcription. Nature 414, 317–322. 11713532 10.1038/35104575

[bib47] Yao J.Y., Xu S., Sun Y.N. et al. (2022). Novel CDK9 inhibitor oroxylin A promotes wild-type p53 stability and prevents hepatocellular carcinoma progression by disrupting both MDM2 and SIRT1 signaling. Acta Pharmacol. Sin. 43, 1033–1045. 34188177 10.1038/s41401-021-00708-2 PMC8975870

[bib48] Yik J.H., Chen R., Nishimura R. et al. (2003). Inhibition of P-TEFb (CDK9/cyclin T) kinase and RNA polymerase II transcription by the coordinated actions of HEXIM1 and 7SK snRNA. Mol. Cell 12, 971–982. 14580347 10.1016/s1097-2765(03)00388-5

[bib49] Yokoyama A., Lin M., Naresh A. et al. (2010). A higher-order complex containing AF4 and ENL family proteins with P-TEFb facilitates oncogenic and physiologic MLL-dependent transcription. Cancer Cell 17, 198–212. 20153263 10.1016/j.ccr.2009.12.040 PMC2824033

[bib50] Yu G., Wang L.G., He Q.Y. (2015). ChIPseeker: an R/Bioconductor package for ChIP peak annotation, comparison and visualization. Bioinformatics 31, 2382–2383. 25765347 10.1093/bioinformatics/btv145

[bib51] Yue J., Dai Q., Hao S. et al. (2021). Suppression of the NTS–CPS1 regulatory axis by AFF1 in lung adenocarcinoma cells. J. Biol. Chem. 296, 100319. 33493519 10.1016/j.jbc.2021.100319 PMC7949158

[bib52] Zhang Y., Liu T., Meyer C.A. et al. (2008). Model-based analysis of ChIP–Seq (MACS). Genome Biol. 9, R137. 18798982 10.1186/gb-2008-9-9-r137 PMC2592715

[bib53] Zheng B., Aoi Y., Shah A.P. et al. (2021). Acute perturbation strategies in interrogating RNA polymerase II elongation factor function in gene expression. Genes Dev. 35, 273–285. 33446572 10.1101/gad.346106.120 PMC7849361

[bib54] Zhou Q., Li T., Price D.H. (2012). RNA polymerase II elongation control. Annu. Rev. Biochem. 81, 119–143. 22404626 10.1146/annurev-biochem-052610-095910 PMC4273853

