# Supplementary material for: Distinct roles of two SEC scaffold proteins, AFF1 and AFF4, in regulating RNA polymerase II transcription elongation
Source: J Mol Cell Biol. 2023 Aug 1;15(8):mjad049. doi: 10.1093/jmcb/mjad049 (PMC11113081; doi:10.1093/jmcb/mjad049)
Supplement: mjad049_Supplemental_File [file mjad049_supplemental_file.pdf]

Supplementary Figures

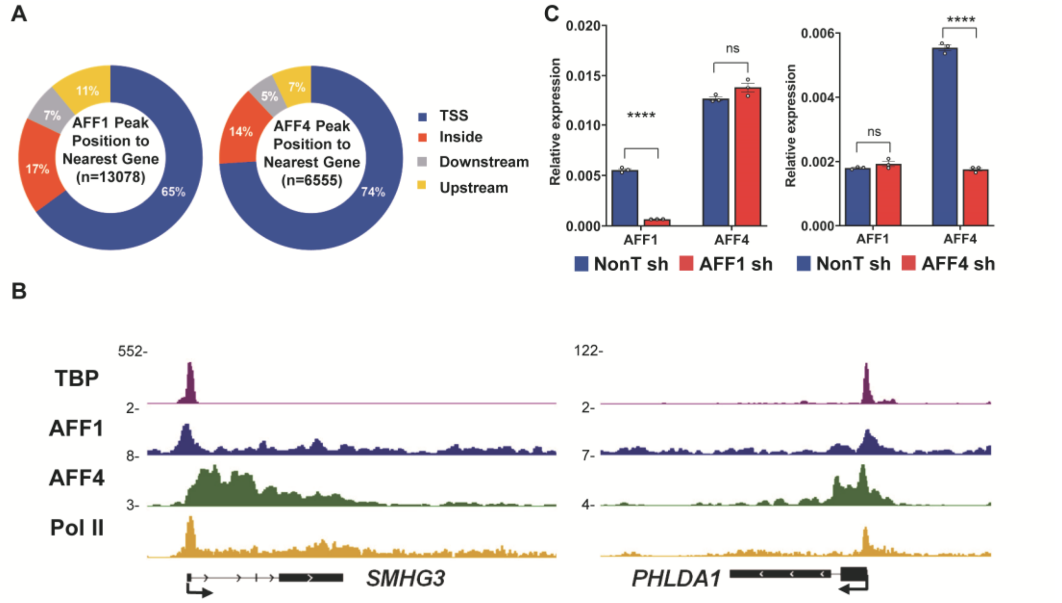

Supplementary Figure S1. AFF1 and AFF4 exhibit diverse chromatin occupancy to regulate Pol II transcription differentially. (A) Pie charts showing that the distribution percentages of AFF1 or AFF4 peaks at different genome locations including TSS, within a gene, and upstream or downstream of the nearest gene. (B) Genome browser track examples for the TBP, AFF1, AFF4 and Pol II ChIP-seq signals. (C) RT-qPCR analyses showing the *AFF1* and *AFF4* knockdown efficiency as well as the mRNA levels of AFF1 and AFF4 were not affected by each other. Expression levels were normalized to *GAPDH* (Each bar represents the mean ± SEM of 3 biological replicates, $*p<0.05$, $**p<0.01$, $***p<0.001$, $****p<0.0001$, ns=not significant., *t* test).

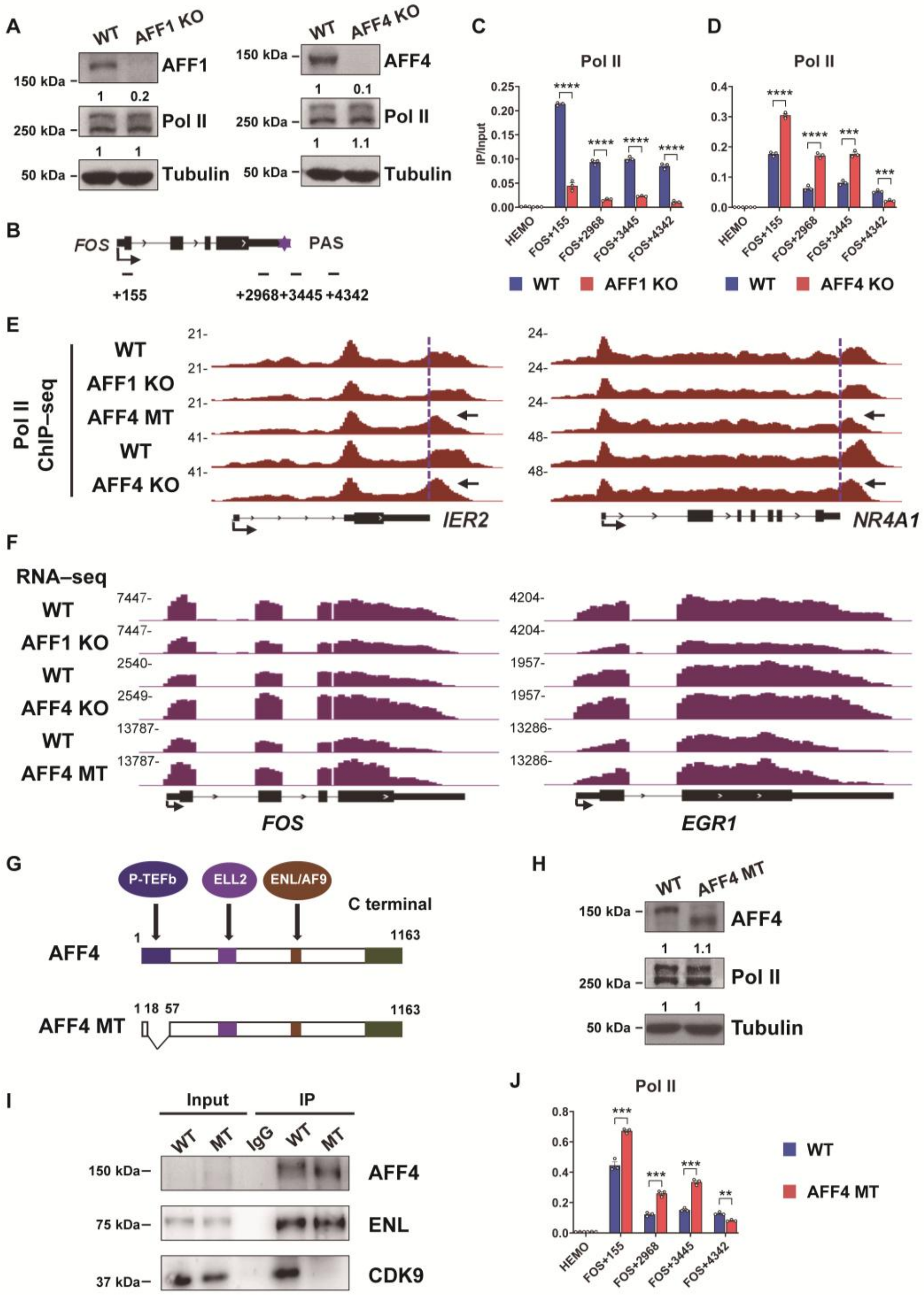

Supplementary Figure S2. AFF4 mutation leads to early termination. (A) Western blot analyses showing the protein levels of AFF1 and Pol II in AFF1 knockout HCT-116 cells as well as the protein levels of AFF4 and Pol II in AFF4 knockout HCT-116 cells. α-Tubulin was used as a loading control. (B) Schematic diagram shows the primers sites around *FOS* gene. (C-D) ChIP-qPCR analysis showing that the Pol II occupancy change at *FOS* gene in control, AFF1 KO and

AFF4 KO cells (Each bar represents the mean $\pm$ SEM of 3 biological replicates, $*p < 0.05$, $**p < 0.01$, $***p < 0.001$, $****p < 0.0001$, ns=not significant., *t* test). (E) Genome browser track examples of Pol II occupancy in control, AFF1 KO, AFF4 KO and AFF4 MT cells. Purple vertical dotted lines denote the TES and black arrows indicate Pol II peak shift towards 5' ends. (F) Two individual gene tracks of RNA-seq in WT, AFF1 KO, AFF4 KO and AFF4 MT are shown. (G) Schematic diagrams indicate AFF4 interaction regions and the deletion region of AFF4 MT cell. (H) Western blot analyses showing the protein levels of AFF4 and Pol II in AFF4 mutation HCT-116 cells. α-Tubulin was used as a loading control. (I) Western blot analysis of AFF4, ENL and CDK9 in the wild type and AFF4 mutation HCT-116 cells through AFF4 immunoprecipitation. (J) ChIP-qPCR analysis showing that the Pol II occupancy change at *FOS* gene in control and AFF4 MT cells (Each bar represents the mean $\pm$ SEM of 3 biological replicates, $*p < 0.05$, $**p < 0.01$, $***p < 0.001$, $****p < 0.0001$, ns=not significant., *t* test).

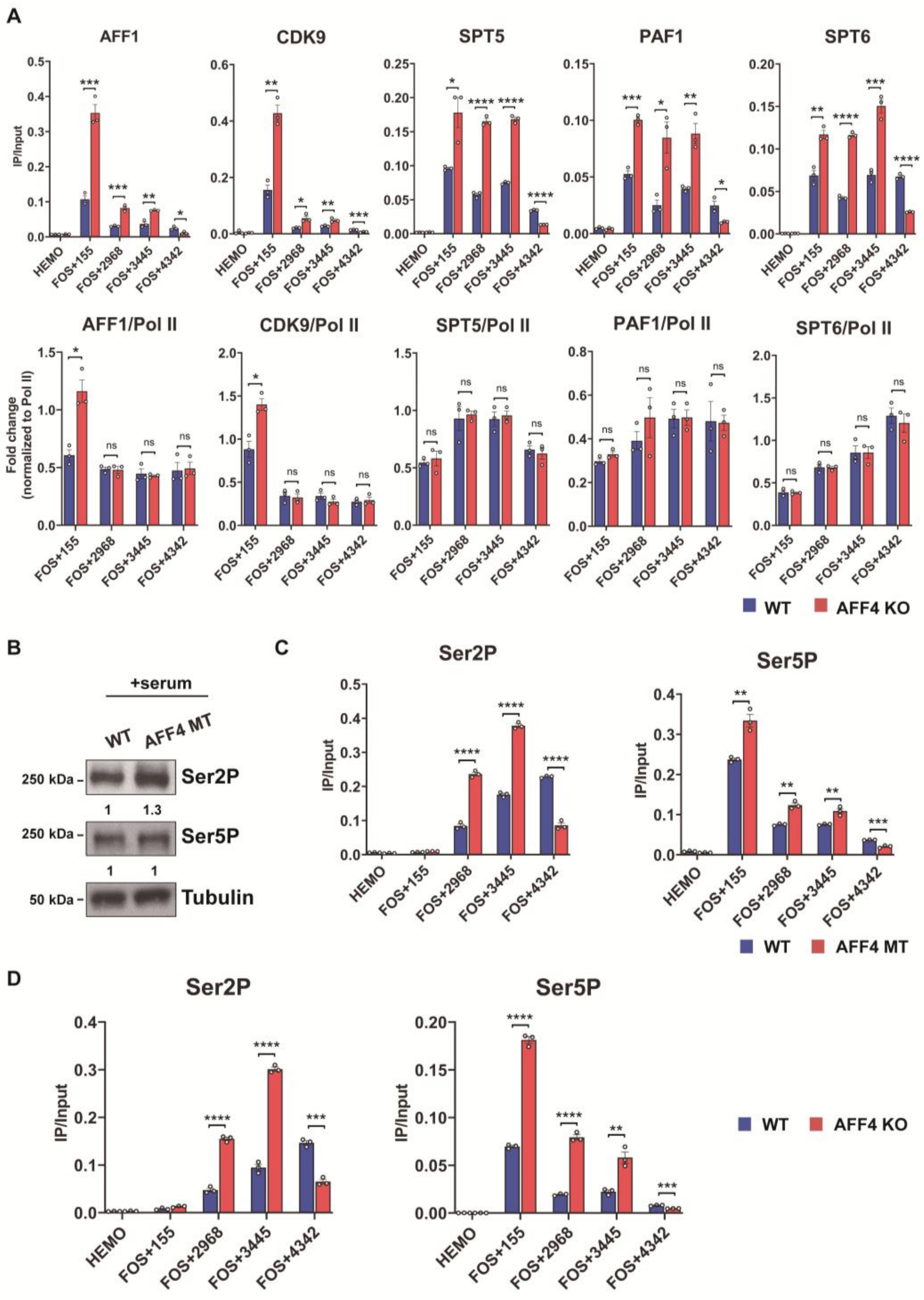

Supplementary Figure S3. AFF4 mutation results in the accumulation of Ser2P, Ser5P and elongation associated factors at 5’ end of genes. (A) ChIP-qPCR analysis showed the occupancies and normalized occupancies of elongation factors (AFF1, CDK9, SPT5, PAF1 and SPT6) at the *FOS* gene in AFF4 KO serum induced HCT-116 cells (Each bar represents the mean ± SEM of 3 biological replicates, * $p < 0.05$, **$p < 0.01$, ***$p < 0.001$, ****$p < 0.0001$, ns=not significant., *t* test). (B)

The protein levels of Pol II Ser2P and Pol II Ser5P in AFF4 MT HCT-116 cells under serum induction. α-Tubulin was used as a loading control. (C) ChIP-qPCR analysis showing that the Pol II Ser2P and Pol II Ser5P occupancy change at the *FOS* gene in control and AFF4 MT serum induced HCT-116 cells. (D) ChIP-qPCR analysis showing that the Pol II Ser2P and Pol II Ser5P occupancy change at the *FOS* gene in control and AFF4 KO serum induced HCT-116 cells (Each bar represents the mean ± SEM of 3 biological replicates, $*p < 0.05$, $**p < 0.01$, $***p < 0.001$, $****p < 0.0001$, ns=not significant., *t* test).

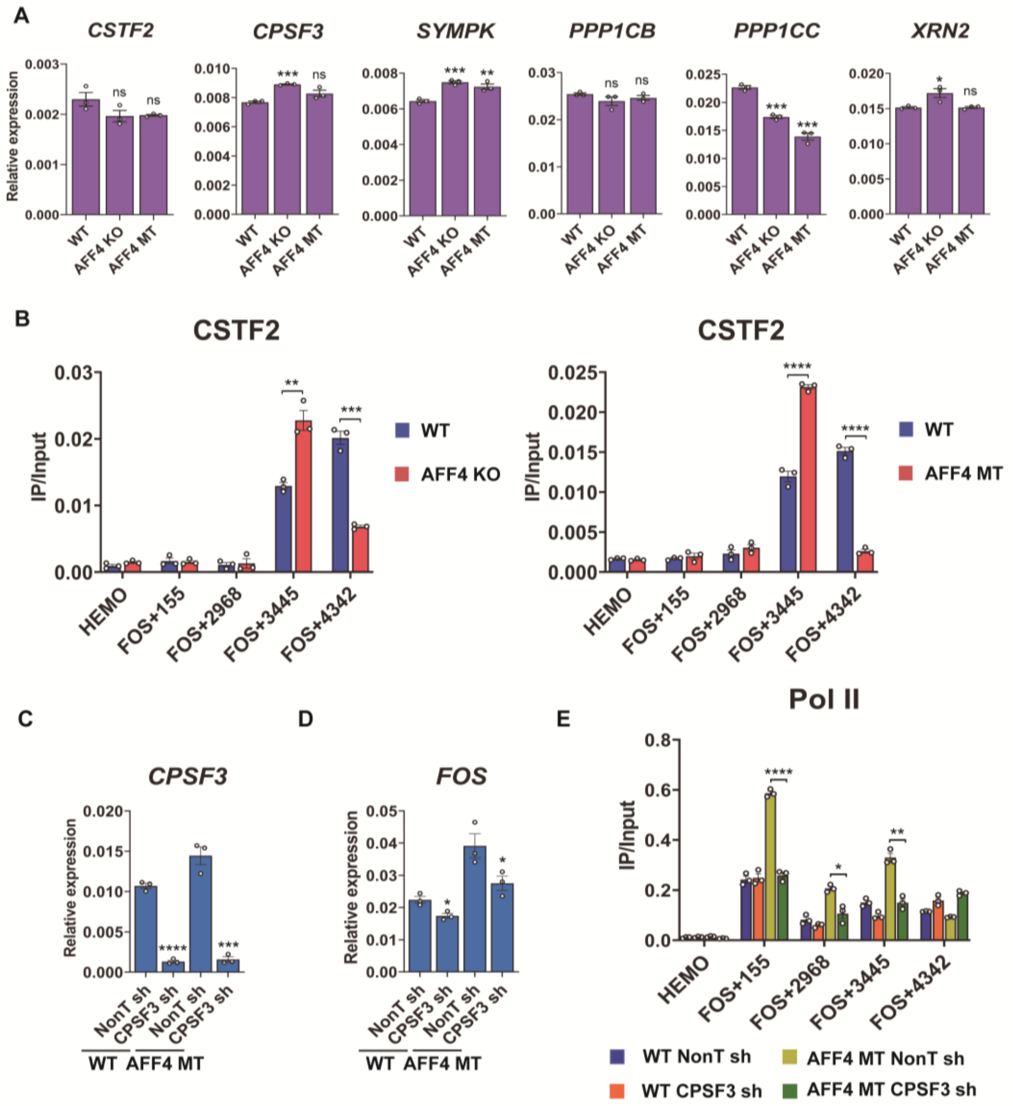

Supplementary Figure S4. AFF4 disruption leads to increased CSTF2 occupancy with a 5' shift. (A) RT-qPCR showing the RNA expression level of *CSTF2*, *CPSF3*, *SYMPK*, *PPP1CB*, *PPP1CC* and *XRN2* in wildtype and AFF4 KO/MT cells. (B) ChIP-qPCR analysis showing the occupancy of CSTF2 at *FOS* gene in AFF4 depletion and AFF4 mutation serum induced HCT-116 cells. The *HEMO* gene acts as a negative control for ChIP-qPCR. (C) RT-qPCR showing the RNA expression level of *CPSF3* after CPSF3 knockdown in wildtype and AFF4 MT cells. (D) RT-qPCR showing the RNA expression level of *FOS* after CPSF3 knockdown in wildtype and AFF4 MT cells. (E) The change of Pol II enrichment around the *FOS* gene after knockdown CPSF3 in WT and AFF4 MT cells analyzed by ChIP-qPCR. The *HEMO*

gene acts as a negative control for ChIP-qPCR (Each bar represents the mean $\pm$ SEM of 3 biological replicates, $*p < 0.05$, $**p < 0.01$, $***p < 0.001$, $****p < 0.0001$, ns=not significant., *t* test).

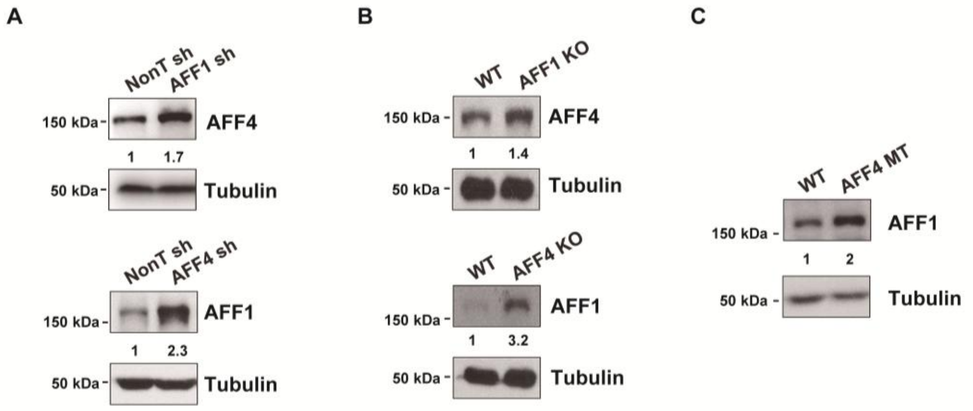

Supplementary Figure S5. Significant increases of AFF4 occupancies at a subset of highly active genes in AFF1-depleted cells. (A) Western blot analyses showing the protein level change of AFF4 in AFF1 knockdown and AFF1 in AFF4 knockdown A549 cells. α-Tubulin was used as a loading control. (B) Western blot analyses showing the protein level change of AFF4 in AFF1 knockout and AFF1 in AFF4 knockout HCT-116 cells. α-Tubulin was used as a loading control. (C) Western blot analyses showing the protein level change of AFF1 in AFF4 MT HCT-116 cells. α-Tubulin was used as a loading control.
